# CeFeO_3_–CeO_2_–Fe_2_O_3_ Systems: Synthesis by Solution Combustion Method and Catalytic Performance in CO_2_ Hydrogenation

**DOI:** 10.3390/ma15227970

**Published:** 2022-11-11

**Authors:** Anna N. Matveyeva, Shamil O. Omarov, Marianna A. Gavrilova, Dmitry A. Sladkovskiy, Dmitry Yu. Murzin

**Affiliations:** 1 Laboratory of Materials and Processes for Hydrogen Energy, Ioffe Institute, Politekhnicheskaya ul. 28, 194021 St. Petersburg, Russia; 2 Resource-Saving Department, St. Petersburg State Institute of Technology (Technical University), Moskovskiy pr. 26, 190013 St. Petersburg, Russia; 3 Laboratory of Industrial Chemistry and Reaction Engineering, Åbo Akademi University, Henriksgatan 2, 20500 Turku, Finland

**Keywords:** perovskite, cerium orthoferrite, CeFeO_3_, solution combustion synthesis, hydrogenation, CO_2_, glycine, urea, urotropine

## Abstract

Rare-earth orthoferrites have found wide application in thermocatalytic reduction-oxidation processes. Much less attention has been paid, however, to the production of CeFeO_3_, as well as to the study of its physicochemical and catalytic properties, in particular, in the promising process of CO_2_ utilization by hydrogenation to CO and hydrocarbons. This study presents the results of a study on the synthesis of CeFeO_3_ by solution combustion synthesis (SCS) using various fuels, fuel-to-oxidizer ratios, and additives. The SCS products were characterized by XRD, FTIR, N_2_-physisorption, SEM, DTA–TGA, and H_2_-TPR. It has been established that glycine provides the best yield of CeFeO_3_, while the addition of NH_4_NO_3_ promotes an increase in the amount of CeFeO_3_ by 7–12 wt%. In addition, the synthesis of CeFeO_3_ with the participation of NH_4_NO_3_ makes it possible to surpass the activity of the CeO_2_–Fe_2_O_3_ system at low temperatures (300–400 °C), as well as to increase selectivity to hydrocarbons. The observed effects are due to the increased gas evolution and ejection of reactive FeO_x_ nanoparticles on the surface of crystallites, and an increase in the surface defects. CeFeO_3_ obtained in this study allows for achieving higher CO_2_ conversion compared to LaFeO_3_ at 600 °C.

## 1. Introduction

The limited reserves of fossil crude oil, as well as global warming, and possible consequences for human and environmental safety, are the driving forces for the development of more environmentally friendly and sustainable processes [1]. Among the technological challenges requiring aspectual attention at present are the valorization of biomass, water splitting, CO_2_ reduction, abatement of exhaust gases, and production of renewable hydrogen [2,3], which has the potential to replace conventional fossil fuels as a clean energy carrier.

The key to solving and overcoming these challenges is catalysis in combination with solid-state chemistry, which is also used to develop tailor-made catalytic materials, and materials science to elucidate the structure-property relationships in defect solids [4]. Subsequently, many materials that have properties important for successful applications in electronics and other fields, such as crystallinity, particle size, porosity, structure defectiveness, and composition variability, can also be effective catalysts.

In particular, perovskites with their unique physical properties are among such materials that are attracting attention for many materials science applications [5], including rechargeable batteries [6], supercapacitors [7], photovoltaics [8], photocatalysts [9], and electrocatalysts [10]. Due to the wide range of ions and valences that this structure can contain, as well as the ability to fine-tune perovskites for specific catalytic requirements, these oxides can be successfully applied as catalytic materials [4].

The term perovskite is used to refer to any compound that has a formula ABX_3_ and for which the B ion is surrounded by an octahedron of X ions, while the original mineral (perovskite) has the chemical formula CaTiO_3_ [5].

Oxides with the perovskite structure have a cubic unit cell, where the A ion is at the center of the unit cell, the B ions are located at the corners, and the negative ions occupy the face-centered positions [5,11]. Therefore, “A” ions are large cations and usually belong to the group of alkali or rare earth metals that fit into the dodecahedral sites of the framework and provide structural stability, whereas “B” ions are smaller cations, typically 3d, 4d, or 5d transition metals, occupying octahedral positions of the framework and undergoing redox transformations [12,13]. Generally, in the perovskite framework, the B-site is the catalytically active site, while the A-site plays a major support role [12].

However, most perovskites are deformed due to steric constraints caused by differences in the ionic radii leading to the formation of oxygen/cationic vacancies and changing angles between cations and oxygen. The stability of perovskites relative to other structures is frequently defined in terms of the Goldschmidt tolerance factor (*t*), the value of which depends on the radii of the cations included in the structure [14,15]. A more detailed description of the structure of perovskites, their various deformations, as well as a definition of their stability, is given in [16,17,18,19,20].

One of the important groups of the perovskite family is rare-earth ferrites, which have recently attracted an increased interest due to the multifunctional properties of many representatives of this class, and the catalysts based on them, are currently used mainly in reduction-oxidation processes [12,13]. LaFeO_3_, among other ferrites, is the most investigated perovskite, which, due to the partially reduced state of near-surface Fe ions (Fe^2+^/Fe^3+^), selectively activates the C-H bond in methane to produce high-purity syngas [13]. For La ferrite, three different types of active iron sites were found, from the point of view of their different surroundings by surface oxygen ions and oxygen vacancies, which determine the completeness of CH_4_ oxidation. Unlike lanthanum, cerium ions are characterized by two charge states (Ce^3+^/Ce^4+^), which, in combination with Fe^3+^/Fe^2+^, suggests that the catalyst based on CeFeO_3_ can be more active in redox processes than LaFeO_3_.

Cerium ferrite is still one of the least investigated representatives of this class, although it has the highest abundance in nature among the lanthanide elements [21]. For CeFeO_3_, an orthorhombic structure of GdFeO_3_ type is usually expected, because *t* is 0.85 [20]. Cerium oxide in this perovskite has a possibility of reversible transformation between Ce^3+^ and Ce^4+^ [22]. Increasing the fraction of Ce^4+^ leads to a decrease in oxygen vacancies and in the oxidation state of Fe due to charge neutrality in CeFeO_3_. The presence of Ce^4+^ and Fe^2+^ slightly distorts the FeO_6_ octahedra and increases the tolerance factor to ca. 0.87.

Very few publications addressed the synthesis of CeFeO_3_, which was structurally described for the first time in [23] because the preparation of CeFeO_3_ is not as simple as that of other rare earth orthoferrites [24]. Initially, Ce orthoferrites were obtained by firing pressed tablets from a mixture of Fe_2_O_3_, CeO_2_, and metallic Fe (as a reducing agent) in a sealed evacuated quartz tube at 800–850 °C for 48 h [25], and under a controlled low partial pressure of O_2_ [26]. This method requires a long reaction time, high temperatures, and a complex experimental setup. However, due to the small ionic radius of the Ce^3+^ cation [21], therefore, CeFeO_3_ is poorly stable in the presence of oxygen and at elevated temperatures. Furthermore, cerium (III) compounds generally tend to oxidize easily to Ce(IV) species, mostly forming stable CeO_2_.

Other synthesis methods have also been proposed, including the co-precipitation method [22,27,28,29] and sol-gel technique [30,31], which are also complex and/or require high calcination temperatures (550–1000 °C) and long reaction time (3–48 h). At the same time, there are difficulties in the targeted synthesis of nanostructured CeFeO_3_ by these methods.

Thus, taking into account the conceptual difficulties to obtain CeFeO_3_, a feasible alternative is highly desirable. The solution combustion synthesis (SCS) method was explored in this study, as it exhibits such advantages as the short reaction time and the concomitant formation of an inert gas, which prevents oxidation of Ce (III). Overall, SCS is one of the simplest, fast, and most efficient methods for the synthesis of both simple and complex metal oxides [32,33] allowing the production of high-purity homogeneous powders [34].

SCS was proposed by Kingsley and Patil [35] as an alternative to self-propagating high-temperature synthesis (SHS) developed in 1967 by Merzhanov, Shkiro, and Borovinskaya [36]. Before the combustion process, the reactants (salts of metal and organic fuel) are brought into solution, ensuring their uniform distribution. The redox mixture, after evaporation of the solvent, ignites and eventually burns out in a self-sustaining and fast combustion reaction [37].

The fundamental possibility of obtaining CeFeO_3_ nanocrystals by SCS was shown in [21,24,37], where glycine or PVA/urea was used as fuel. However, only in some studies was it possible to obtain CeFeO_3_ without a noticeable amount of impurity phases. Quantitative phase analysis performed in [24] revealed that the product obtained using glycine-nitrate combustion (fuel-to-oxidizer ratio (φ) of 1.5) consisted of CeFeO_3_ with a minor amount of CeO_2_ (6.8 wt%). In [21], the optimal fuel-to-oxidizer ratio at which CeFeO_3_ is formed with the minimum amount of Fe_2_O_3_ (ca. 14 wt%) and CeO_2_ (ca. 30 wt%) was also found to be 1.5.

In this work, the possibility of CO_2_ hydrogenation using CeFeO_3_-containing catalysts prepared by SCS was investigated. CO_2_ hydrogenation to CO via the reverse water–gas shift reaction (RWGS, CO_2_ + H_2_ → CO + H_2_O) is one of the most promising processes of CO_2_ utilization because CO can be used in the downstream Fischer–Tropsch synthesis (FTS) and other applications [38,39,40,41]. The RWGS reaction is also of interest in the context of human missions to Mars, primarily because of its ability to produce water, which is a source of oxygen [42].

The effect of process conditions (fuel, fuel-to-oxidizer ratio, additives) on the physicochemical and catalytic properties of the synthesized materials was also investigated. To highlight the importance and potential of the synthesis method for perovskite-based catalysts, the catalytic efficiency was compared with the literature data.

## 2. Materials and Methods

### 2.1. Reagents

Ce(NO_3_)_3_·6H_2_O (99.8%, Chemcraft, Kaliningrad, Russia), Fe(NO_3_)_3_·9H_2_O (98.3%, Lenreactiv, St. Petersburg, Russia), NH_4_NO_3_ (98.5%, Russia), glycine (C_2_H_5_NO_2_, 99.2%, Lenreactiv, St. Petersburg, Russia), glucose (C_6_H_12_O_6_, 99.8%, Lenreactiv, St. Petersburg, Russia), urea ((NH_2_)_2_CO, 99.8%, Lenreactiv, St. Petersburg, Russia), and urotropine ((CH_2_)_6_N_4_, 99.5%, Russia) were used.

### 2.2. Synthetic Procedures

For the preparation of cerium orthoferrite CeFeO_3_, an appropriate amount of the fuel (glycine, glycine-glucose, urea, or urotropine) and the corresponding nitrates were dissolved in deionized water (1 mL of water per 1 g of the starting materials) in a 250 mL wide glass beaker. The weight of the reagents was taken to ensure the synthesis of 1 g of CeFeO_3_. Ammonium nitrate was used in the amounts of 1 g per 1 g of perovskite, while the molar ratio of glucose to glycine was 1:6. The fuel-to-oxidizer ratio (φ) was taken from 1 to 3.7 and calculated according to the following equation [34]:

φ = (−1)Σ(coeff.∙RV of fuel)/Σ(coeff.∙OV of nitrate), (1)

where RV—the reducing valency, OV—the oxidizing valency.

In this method, metals, “C” and “H” are considered reducing elements with their corresponding valence, +4 for carbon and +1 for hydrogen. Oxygen and nitrogen are considered oxidizing agents with the valences −2 and 0, respectively [43].

For example, the reaction equation occurring in the process can be represented as:

(2) $$4\mathrm{Ce}{\left({\mathrm{NO}}_{3}\right)}_{3}(\mathrm{aq})\;+\;4\mathrm{Fe}{\left({\mathrm{NO}}_{3}\right)}_{3}(\mathrm{aq})\;+\;20C_{2}H_{5}{\mathrm{NO}}_{2}\;+\;15O_{2}\to \;4\mathrm{Ce}\mathrm{Fe}O_{3}\;+\;40{\mathrm{CO}}_{2}\;+\;22N_{2}\;+\;(50H_{2}O)$$

According to this equation, the reducing valence of glycine (C_2_H_5_NO_2_) is +9 (RV = 4∙2 + 1∙5 + 0 – 2∙2), whereas the oxidizing valence of both Ce(NO_3_)_3_ and Fe(NO_3_)_3_ is equal to –15 (OV = 3 – 0 – 2∙3∙3), therefore φ is 1.5 (–1·9·20/(–15·4 + (–15)·4).

The obtained solution was heated to boiling on an electric plate (1 kW) and, after complete evaporation of water, was burned with the formation of a solid. Two-three batches of each material were made. Reaction equations for each synthesis are presented in the Supplementary Material.

### 2.3. Characterization

XRD analysis was done using a SmartLab 3 Diffractometer (Rigaku, Japan) equipped with Ni-filter (Rigaku, Japan) and 1D detector (DteX250, Rigaku, Japan) at the following conditions: CuKα radiation (λ = 1.54056 Å), 40 kV, 30 mA, a scan speed of 4°/min, and a step width of 0.01°. To determine the phase composition and the crystal structural parameters, the diffraction data were analyzed by the Rietveld method. PDWin software (v. 2.0, 2004) was used for the refinement. The structural parameters for CeFeO_3_ with the space group Pbnm, CeO_2_ with the space group Fm$\overset{-}{3}$m, and Fe_2_O_3_ with the space group Fd$\overset{-}{3}$mZ were taken using the ICDD PDF-2-2016 database (No. 93611, 193169, and 247036, respectively). The Pseudo-Voigt function was used for the simulation of the peak shape. The background was modeled by a Chebyshev polynomial. The mean crystallite size was calculated using the Scherrer equation.

An infrared transmission spectrum was determined using a IRTracer-100 spectrophotometer (Shimadzu, Japan). The IR spectra were recorded from 4000 to 350 cm^−1^. One-two mg of each solid sample was mixed with ca. 200 mg of vacuum-dried IR-grade KBr. The mixture was dispersed by grinding in an agate mortar, placed in a steel die 13 mm in diameter, and gradually subjected to a pressure of 12 tons under vacuum pumping.

N_2_-physisorption analysis was performed on ASAP 2020 (Micromeritics, Norcross, GA, USA) and Autosorb-6iSA (Quantachrome Instruments, Boynton Beach, FL, USA). Prior to the measurements, the samples were degassed at 200 °C under vacuum. The specific surface area and the total pore volume were determined from N_2_ adsorption isotherms applying the BET equation. The pore size distribution was calculated by the DFT (Density Functional Theory) method.

SEM was performed in SE mode using a VEGA 3 SBH microscope (TESCAN, Czech Republic) equipped with Oxford instruments INCAx–act for energy-dispersed X-ray spectroscopy (EDS).

H_2_-TPR was performed on Chemosorb (SOLO, Novosibirsk, Russia) with a thermal conductivity detector (TCD). Before H_2_-TPR, an SCS-product (ca. 60 mg) was pretreated in air to ca. 330–350 °C. The reduction was performed from 50–100 to 900 °C at a ramp rate of 10°/min under 10 vol. % H_2_ in Ar (99.998 vol. % purity) and a total gas flow of 20 mL/min. Isopropanol cooled in liquid nitrogen was used as a water trap. CuO was used to calibrate hydrogen consumption.

TGA and DTA were done on a simultaneous thermal analyzer DTG-60A (Shimadzu) by heating a sample in air or nitrogen to 800 °C with a rate of 10 °C/min. The weight measurement error was 1% for TGA and 1 μV for DTA.

### 2.4. Catalytic Tests

The experiments were carried out in a fixed-bed reactor at atmospheric pressure. Approximately 300 mg of the powder sample (the fraction below 100 μm) was placed on a diffuser grid made of SiO_2_ in the cylindrical quartz reactor (37 mm in length, 1.5 cm in inner diameter), which contained a thermocouple pocket with a diameter of 5 mm. The gases were fed into the reactor from top to bottom. The sample was initially heated in the air flow (30 mL/min) to 300 °C, then flushed with N_2_ (99.999 vol. % purity, 20 mL/min) up to 400 °C. Thereafter, 50 vol. % H_2_ in N_2_ was fed with a total flow of 40 mL/min for 1 h. After reduction, the sample was exposed to the reactants at a weight hourly space velocity (WHSV) of 10,000 mL·g^–1^·h^–1^ (30 mL/min H_2_, 10 mL/min CO_2_, and 10 mL/min N_2_) for 15 min consecutively at 300, 400, 500, and 600 °C. Nitrogen in the gas flow was used as the internal standard for gas chromatography, allowing the establishment of mass balance. In order to prevent water from entering the GC, the outlet pipe passed through a cooling trap.

For comparison, long-term tests were performed at an elevated WHSV (72,000 mL·g^–1^·h^–1^), and a stoichiometric H_2_/CO_2_ ratio was chosen to evaluate the CO_2_ utilization efficiency. After reduction, the sample was exposed to RWGS feed (40 mL/min H_2_, 40 mL/min CO_2_, and 40 mL/min N_2_) at 600 °C for 4 h.

The gas products were analyzed on a GC-2010 Plus chromatograph (Shimadzu) with a TCD (RT-Msieve 5A capillary column (30 m, dinner = 0.53 mm) and Rt-Q-BOND capillary column (30 m, dinner = 0.53 mm); temperature program: 30 °C–5 min; 30–60 °C with a heating rate 4°/min; 60–100 °C with a heating rate 15°/min; 100–150 °C with a heating rate 30°/min; 150 °C–3 min; 150–180 °C with a heating rate 5°/min; 180 °C–23.9 min).

Catalytic behavior was evaluated in terms of CO_2_ conversion (*X*, %), selectivity to CO and methane ($S_{CH_{4}}$, mol%). The following equations were used for calculating:

(3) $$X_{CO_{2}}=\frac{Molar\;flow\;C\;from\;products}{Molar\;flow\;C\;from\;CO_{2}+products}\cdot 100\%$$

(4) $$S_{i}=\frac{Molar\;flow\;C\;from\;i}{Total\;molar\;flow\;C\;from\;products}\cdot 100\%$$

where *i*–CO, CH_4_, C_2_H_4_, C_2_H_6_, C_3_H_8_; molar flow C from *i*–molar flow of the product multiplied by the number of carbon atoms in the compound, mol/h; total molar flow C = *n*(CO) + n(CH_4_) + 2·*n*(C_2_H_4_) + 2·*n*(C_2_H_6_)+3·*n*(C_3_H_8_), mol/h.

The deactivation rate (*k_d_*) with time-on-stream (*t*) was calculated with the assumption that the deactivation follows first-order kinetics as follows:

(5) $$k_{d}=\frac{1}{t}(\ln\frac{1-{\left(X_{CO_{2}}\right)}_{t}}{{\left(X_{CO_{2}}\right)}_{t}}-\ln\frac{1-{\left(X_{CO_{2}}\right)}_{0}}{{\left(X_{CO_{2}}\right)}_{0}})$$

where ${\left(X_{CO_{2}}\right)}_{0}$ is the initial conversion of CO_2_; ${\left(X_{CO_{2}}\right)}_{t}$–the conversion of CO_2_ at time *t*.

The rate of CO_2_ hydrogenation (*r*, mmol·s^–1^·g^–1^) was calculated using the following equation:

(6) $$r=\frac{X_{CO_{2}}\cdot F_{CO_{2}}}{m_{cat}}$$

where *F*–the CO_2_ flow rate (mmol/s), *m_cat_* is the catalyst weight (g).

## 3. Results and Discussion

### 3.1. Synthesis Features and Phase Composition

**Figure 1:**
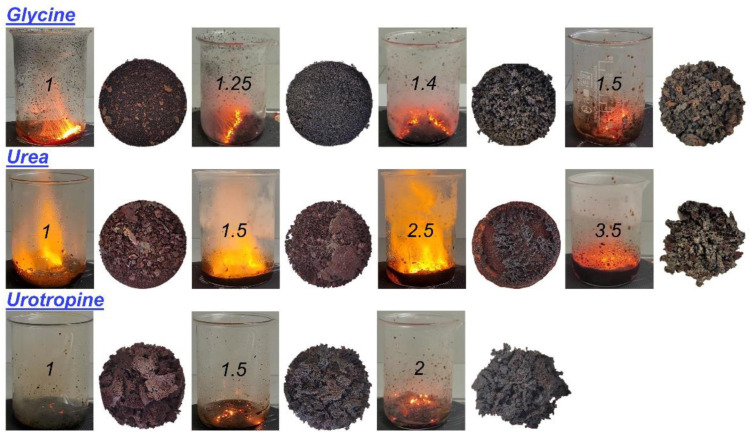
The course of the fuel-nitrate solution combustion and the macro morphology of the SCS products obtained at various fuel-to-oxidizer ratios.

Based on the previous studies on the synthesis of CeFeO_3_ with the solution combustion method [21,24], the first syntheses were carried out using glycine as fuel with a fuel-to-oxidizer ratio of 1–1.5. Figure 1 shows the visual course of the combustion and the macro morphology of the obtained SCS products.

**Figure 2:**
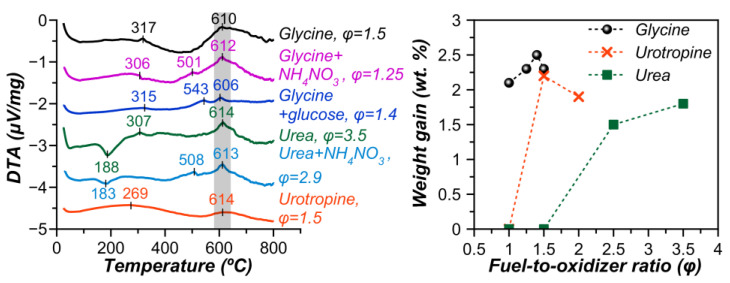
Differential thermal curves and weight gain during heating in air for the as-prepared samples produced by SCS using different fuels, additives, and fuel-to-oxidizer ratios.

It can be clearly seen from Figure 1 that combustion in the specified range of φ proceeds quite efficiently in a self-propagating mode. Abundant gas evolution leads to the formation of the fluffy powders. Despite incremental changes in the fuel-to-oxidizer ratio, the morphology and the color of the obtained solid products have changed significantly. Qualitatively, it is possible to predict the presence of the Fe_3_O_4_ (magnetite) phase in the samples in small amounts, judging by the dominance of the deep black color. The DTA curves (Figure 2), respectively, also indicate the presence of magnetite, which at ca. 300 °C turns into γ-Fe_2_O_3_, and the latter at ca. 600 °C undergoes a transformation into α-Fe_2_O_3_ (hematite) [44]. An increase in the mass of the samples upon heating in air in the range from 300 to 800 °C (Figure 2) indicates the presence of cerium orthoferrite, which undergoes oxidation according to the following reaction [45]: 6CeFeO_3_ + O_2_ = 6CeO_2_ + 2Fe_3_O_4_. The largest weight gain occurs when heating the sample obtained at a fuel (glycine)-to-oxidizer ratio of 1.4.

**Figure 3:**
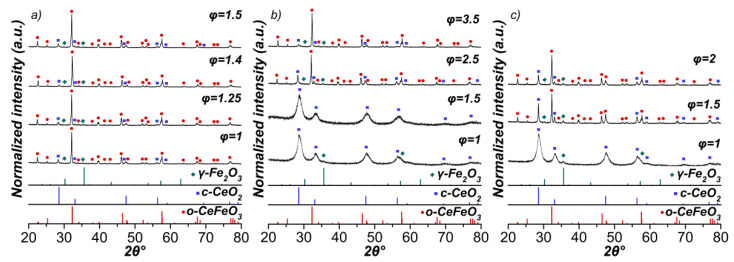
XRD patterns of synthesized materials by (**a**) glycine-nitrate; (**b**) urea-nitrate; and (**c**) urotropine-nitrate combustion at the various fuel-to-oxidizer ratios (φ) (where o-CeFeO_3_–orthorhombic perovskite CeFeO_3_ (JCPDS card no. 00-022-0166); c-CeO_2_–cubic CeO_2_ (JCPDS card no. 01-075-8371); γ-Fe_2_O_3_–cubic Fe_2_O_3_ (JCPDS card no. 00-039-1346)).

XRD measurements prove the successful synthesis of the rare earth orthoferrite CeFeO_3_ by glycine-nitrate solution combustion synthesis (Figure 3a). However, in all cases, there are side phases of γ-Fe_2_O_3_/Fe_3_O_4_, which are difficult to distinguish by XRD analysis, and cubic CeO_2_. It is believed that oxidation of a small amount of Ce(III) to Ce(IV) can occur before the ignition of the solution [24].

**Figure 4:**
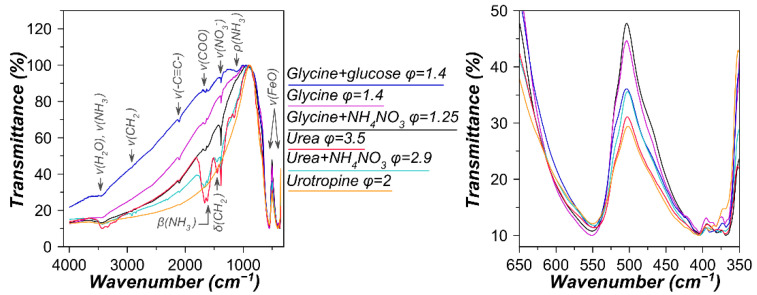
IR spectra of the as-prepared samples produced by solution combustion synthesis using different fuels, additives, and fuel-to-oxidizer ratios (φ).

Compared to glycine, combustion in the presence of urea proceeds with an intensive flame both at a stoichiometric fuel-to-oxidizer ratio and upon an excess of urea φ = 2.5 (Figure 1). The extra heat from the urea combustion promoted sintering through enhanced diffusion. However, only at φ = 2.5 and more, was it possible to obtain the Ce orthoferrite phase (Figure 3b). According to the total weight change (Figure S1), despite the formation of orthoferrite, a very large amount of unburned fuel remained at φ = 3.5. This was also confirmed by IR spectroscopy showing intense bands of functional groups corresponding to the fuel (Figure 4, Table S1).

Unlike glycine and urea, urotropine (hexamethylenetetramine) with lower enthalpy of combustion [46,47] has not been previously used to obtain cerium orthoferrite. No obvious ignition was noticed and combustion proceeded in a smoldering mode (Figure 1). In the case of a stoichiometric ratio, a glass beaker could not resist a large heat release. Nevertheless, at φ = 1.5 and 2, it was possible to synthesize CeFeO_3_ perovskite, despite a higher content of impurity phases compared with the use of other fuels (Figure 3c), Table 1).

For the SCS products containing reflections of cerium orthoferrite, structural studies were carried out with refinement by the Rietveld method. The quantitative phase composition and the average crystallite size (D) are presented in Table 1. For some samples, the Rietveld method was not applicable to calculate the content of Fe_2_O_3_. This suggests that the Fe_2_O_3_ particles are very small and highly dispersed on the surface or the content of free Fe_2_O_3_ is below the detection limit of XRD. Similar data were obtained in [24], where iron oxide was also not taken into account in the composition. The phase composition was corrected considering amorphous iron oxide based on the CeO_2_ content obtained by the Rietveld method. Table S2 contains data to benchmark the reproducibility of the synthesis results from batch to batch. The composition and the size of the orthoferrite crystallites have good reproducibility (4.5 and 6.5%, respectively), while the crystallite size of oxide phases was less efficiently reproduced due to low content of these phases.

The largest amount of CeFeO_3_ (ca. 93 wt%) was obtained using glycine as a fuel at φ = 1.4, while urotropine, on the contrary, led to the lowest content of the target phase. The average size of CeFeO_3_ crystallites ranges from ca. 49 to 75 nm depending on the fuel and its amount, which directly affects the combustion temperature. A higher temperature of combustion during SCS generally results in a larger particle size, stronger agglomeration, and a lower specific surface area of the products. At the same time, higher amounts of released gases increase porosity and the specific surface area. Therefore, the pore structure is a compromise of the combustion temperature, as well as the type and amount of the gaseous products [48,49]. For example, with the same ratio of the fuel-to-oxidizer equal to 1.5, glycine, due to the lower combustion temperature and high gas evolution, afforded more dispersed particles of orthoferrite compared to urotropine (Table 1).

The influence of ammonium nitrate and glucose addition to the fuel on the purity of cerium orthoferrite was also studied (Table S3). Neat ammonium nitrate as a strong oxidizing agent supports and accelerates fuel combustion. It was observed that upon adding 1 g NH_4_NO_3_ to a mixture of glycine with metal nitrates, the amount of formed perovskite increased from ca. 82 (N.4, Table 1) to ca. 94 wt% (N.5). A similar situation occurs during firing with urea when the yield of perovskite increased from 80 to 87 (N.9 and 10, Table 1). This phenomenon can apparently be explained by an increase in the formation of gases that prevent the oxidation of orthoferrite. Such Ce orthoferrite content is the highest obtained by the SCS method among previously published studies (96 versus 93 wt% [24], excluding iron oxide).

Glucose, as a typical carbohydrate, being a large and bulky molecule, tends to leave large voids while burning. Combustion of glycine with glucose is accompanied by the ejection of the sample in the form of fragile filaments (see Table S3). Despite this, the Ce orthoferrite content is greatly reduced from 74 to 63 wt% (N.1 and 7, Table 1).

The size of crystallites in Ce-Fe oxide systems was also analyzed. Reflections of iron oxide are poorly detected (Figure S2), therefore the results in Table 2 are presented for only CeO_2_. The smallest size of ceria was obtained by using glycine and glucose at a high fuel-to-oxidizer ratio, but in this case, almost a third of the fuel was not burned (N.13, Table 2). Urea and urotropine at φ = 1 have led to approximately the same size of crystallites and weight losses.

### 3.2. Structural and Textural Properties

**Figure 5:**
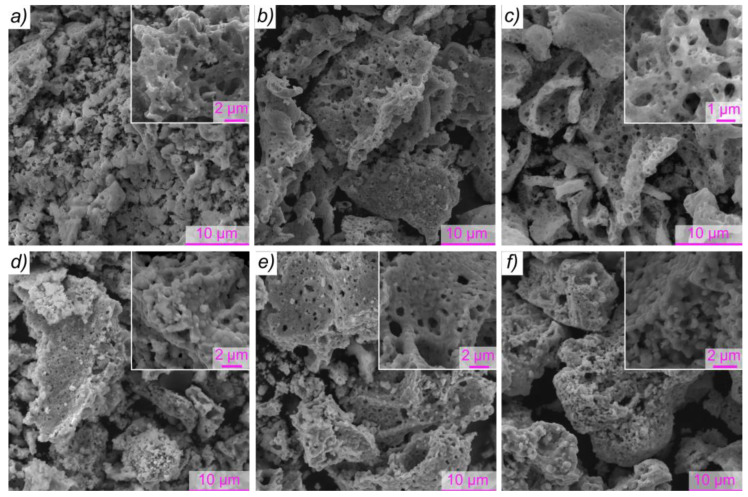
SEM images of the SCS products obtained using different fuels and additives (the upper-right inset shows the corresponding high magnification image): (**a**) glycine, φ = 1.4; (**b**) glycine and NH_4_NO_3_, φ = 1.25; (**c**) glycine and glucose, φ = 1.4; (**d**) urea, φ = 3.5; (**e**) urea and NH_4_NO_3_, φ = 2.9; (**f**) urotropine, φ = 2.

SEM images of the powders prepared using different types of fuel are shown in Figure 5. For all SCS products, micrographs reveal a porous foam-like network of agglomerated interconnected particles. As can be seen, when using glycine (Figure 5a–c), in contrast to urea (Figure 5d,e) and urotropine (Figure 5f), it is not possible to reveal pronounced grain boundaries in the samples, crystals of which have coalesced into each other. This can be attributed to a lower flame temperature reached for the reaction between glycine and nitrates [47].

Fuel type, as well as the fuel-to-oxidizer ratio, can strongly affect closed and open porosity, the average thickness of the pore wall, etc. The most porous sample, as expected, was prepared using glucose as an additive to glycine (Figure 5c). In turn, the dimensional restrictions of the pore walls (a neck) can affect the phase composition of the SCS products. Insufficient pore wall thickness for the formation of an orthoferrite phase stable under the given conditions can be the reason for the formation of more stable products (CeO_2_, Fe_2_O_3_) with smaller crystallites [21]. This explains a decrease in the content and diameter of cerium orthoferrite crystallites upon using glucose (Table 1).

**Figure 6:**
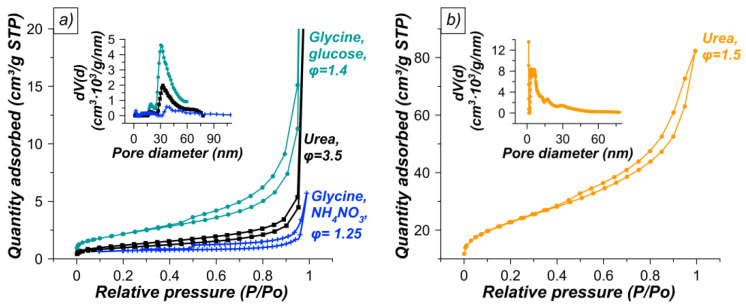
N_2_ adsorption-desorption isotherms and pore size distribution for (**a**) the as-prepared CeFeO_3_–CeO_2_–Fe_2_O_3_ and (**b**) CeO_2_–Fe_2_O_3_ systems produced by SCS using different fuels, additives, and fuel-to-oxidizer ratios (φ).

To estimate the specific surface area (S_BET_) and the total pore volume (ΣV_pore_), low-temperature N_2_ adsorption-desorption was performed, the results of which are presented in Table 3 and Figure 6. S_BET_ for samples with a high content of CeFeO_3_ (80–94 wt%) turned out to be extremely low (2–3 m^2^/g). A sample prepared using glucose as a second fuel allowed a slight increase in the specific surface area to ca. 8 m^2^/g compared to a sample with the same CeFeO_3_ content obtained by urotropine-nitrates combustion (N.12, Table 3). No positive effect of NH_4_NO_3_ on the porosity characteristics of the obtained materials has been found in this study, in contrast to [50], where the use of ammonium nitrate with a mixture of urea and metal nitrates increased the specific surface area of perovskite LaMnO_3_ from 4 to ca. 20 m^2^/g. The sample containing only iron and cerium oxides has a fairly large specific surface area of ca. 81 m^2^/g (N.15, Table 3), which is in fact larger than that of the Ce–Fe oxide system obtained by coprecipitation (14 m^2^/g, [51]).

All SCS products have an IUPAC type IV(a) isotherm [52], which is typical of a mesoporous structure. Apparently, small volumes of mesopores are formed due aggregation of small crystalline particles. The hysteresis loop is of the H4 type, often found in aggregated crystals. The pore size distribution for the perovskite-containing system (Figure 6a) is relatively wide, with maxima of ca. 20 and 30–40 nm compared to the Ce–Fe oxide system, which has a narrower pore size distribution with a maximum of ca. 4 nm (Figure 6b).

### 3.3. Reducibility

**Figure 7:**
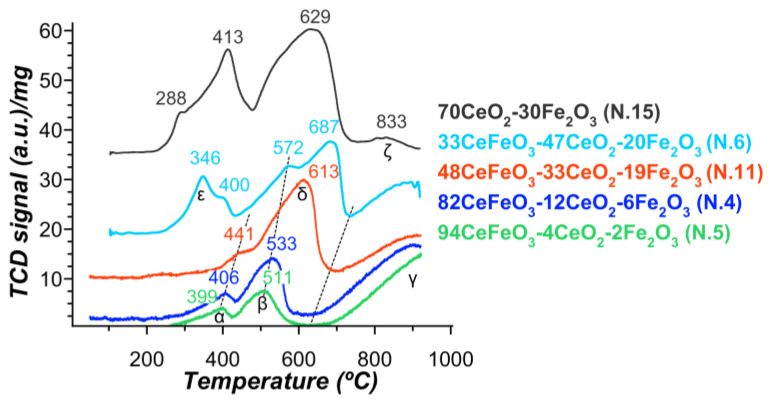
Temperature-programmed reduction (TPR) spectra of the SCS-products calcined at 330–350 °C.

Because reducibility is an important factor influencing the catalytic efficiency of a perovskite, an H_2_-TPR analysis was performed. The reduction process leads to complex TPR profiles and depends on the phase composition, as can be seen in Figure 7. The corresponding amounts of hydrogen consumed in the various reduction steps, as well as the corresponding calculated amounts of hydrogen required for the reduction of Fe_2_O_3_ and CeO_2_, are summarized in Table 4.

For all samples, the presence of free iron oxide was noted, although in different amounts. It is well known that the reduction of Fe_2_O_3_ proceeds via Fe_3_O_4_ (magnetite) and FeO (wüstite) to iron [53,54,55]. The formation of FeO is often not observed since it is metastable and disproportionates into Fe_3_O_4_ and Fe below 620 °C [56]. According to the authors of [53], in the stepwise reduction of iron oxide, the experimental TPR profile of Fe_2_O_3_ contained two peaks at 394 and 545 °C, which correspond to Fe_3_O_4_ and FeO + Fe, respectively. The ratio of the area of the first peak to the area of the second peak should be close to 1:8.

Three reduction steps were observed on the reduction profiles of samples with a high CeFeO_3_ content (48–94 wt%) centered at: 399–441 °C (peak α); 511–613 °C (peak β); and above 650 °C (peak γ), which did not reach the end at 900 °C. The reduction of perovskite CeFeO_3_ mainly occurs at temperatures above 650 °C. In the case of a sample containing 94 wt% CeFeO_3_, the amount of hydrogen consumed in the first two steps is almost three times higher than expected. Thus, it cannot be excluded that surface iron ions from perovskite started to be reduced at lower temperatures. In addition, the ratio of the first two peaks is 0.8. This is due either to an increased amount of Fe^2+^ in free FeO_x_ and the surface of CeFeO_3_, as well as the formation of oxygen-deficient CeO_x_, or the formation of cation-deficient CeFeO_3_. The first leads to an increase in the anion defectiveness of the system. In the second case, changing the number of A or B ions will initially lead to an increase in the charge of the corresponding B or A cations, similar to LaFeO_3_ with a deficiency of La [57]. However, at the stage of reductive activation, as well as during a catalytic process, their reduction and formation of additional oxygen vacancies are expected.

According to the literature [58], iron is mainly present in the +3-oxidation state in an AFeO_3_ perovskite (where A is a rare earth metal), therefore, the following reduction steps can be proposed [59,60]: (1) the partial reduction of Fe^3+^ (clustered and surface) to Fe^2+^; (2) complete reduction of Fe^3+^ (aggregated) to Fe^2+^, with a possible additional consumption of hydrogen for the surface reduction of Fe^2+^; (3) reduction of the remaining Fe^2+^ to Fe^0^ and obtaining individual phases of Ce_2_O_3_ and Fe.

CeO_2_–Fe_2_O_3_ and a sample with a low CeFeO_3_ content (33 wt%, N.6) exhibit similar, although more complex profiles, as two additional peaks δ and ε appear at ~288–346 °C and ~629–687 °C. These peaks can hardly be attributed to cerium oxide, despite its high content. For pure CeO_2_, two characteristic peaks are usually observed at ca. 500 and 800 °C [61,62], which are associated with the reduction of surface and bulk oxygen, respectively. However, the CeO_2_–Fe_2_O_3_ system has only a low-intensity CeO_2_ peak at ca. 800 °C (peak ζ), thus the low-temperature peak should also be of low intensity.

In the case of the Ce–Fe oxide system, CeO_2_ (002) reflection is also shifted toward larger diffraction angles, and the lattice parameters of cerium decrease to 5.376 Å (Table 4), which indicates the formation of a Ce_1-x_Fe_x_O_2-d_ solid solution after the replacement of Ce^4+^ (0.97 Å) to Fe^3+^ with a smaller size (0.64 Å) inside the structure of the cubic phase of CeO_2_. This leads to the formation of oxygen vacancies owing to the charge balance [56,61]. It was shown in [56] that the reduction ability increases with the close interactions of Fe and Ce cations.

Therefore, Fe_2_O_3_ in the Ce–Fe oxide system undergoes a three-stage reduction of Fe_2_O_3_ to Fe_3_O_4_ (413 °C), Fe_3_O_4_ to FeO (ca. 550 °C), and then to Fe (629 °C), as can be confirmed by 2D XRD pattern recorded during H_2_-TPR for 70Ce−Fe [51]. Regarding the peak at ~288–346, it can be associated according to [63] with dispersed Fe.

In summary, the results of the characterization revealed that the phase transformation of the synthesized materials is strongly affected by the amount of perovskite. To evaluate the impact on catalytic properties, the samples with different amounts of CeFeO_3_ were tested in CO_2_ hydrogenation.

### 3.4. Catalytic Performance

**Figure 8:**
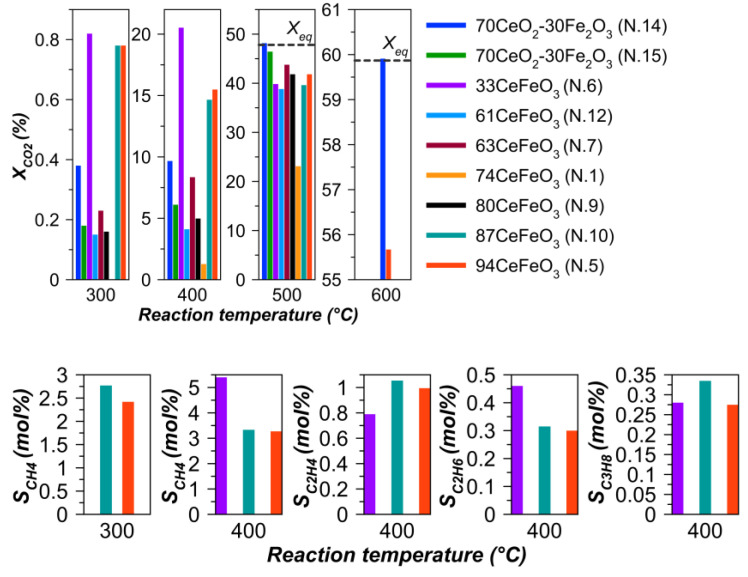
Dependence of CO_2_ conversion and selectivity to hydrocarbons on temperature at H_2_:CO_2_:N_2_ = 3:1:1, WHSV = 10 000 mL·g^–1^·h^–1^. CO selectivity is 100 mol%, except for samples additionally generating hydrocarbons (N. 5, 6, 10).

Figure 8 shows the catalytic behavior of the samples as a function of reaction temperature and perovskite content. As expected, the temperature has a critical effect on CO_2_ conversion. Elevation of temperature resulted in lower apparent activation energy, which can be associated with a change in the kinetic regime through the regime of internal mass transfer limitations to the regime of limitations by external diffusion (Figure S3). It was found that some samples are more active at temperatures of 300–400 °C, while others are more active at 500–600 °C. The observed activation energy for CO_2_ hydrogenation in the range of 300–400 °C was determined to be 101 kJ/mol for the Ce–Fe oxide system (N.14), and 87 kJ/mol for the material containing CeFeO_3_ (N.5). These data generally correspond to the literature data, where the activation energy was reported to be 80 kJ/mol for Fe_3_O_4_ (327–427 °C), and 110 kJ/mol for an industrial catalyst Cu/ZnO/A1_2_O_3_ (170–260 °C) [64].

With increasing the perovskite content no clear trends in conversion could be seen; therefore, it is quite probable that iron oxide, and not only perovskite, is also responsible for the activity of all samples. CO_2_ hydrogenation over Fe-based catalysts has been extensively investigated because they contain active sites necessary to realize the two catalytic reactions in this process: CO production by reverse water gas shift reaction (RWGS) and Fischer–Tropsch synthesis (FTS) with hydrocarbon formation [65].

The dominant product for all samples, and for many even the only one, is CO. For the three most active samples (N.5, 6, 10) at a low reaction temperature, the formation of C_1_–C_3_ hydrocarbons is observed (Figure 8). High RWGS activity and absence of FTS have been found earlier for Fe oxide phases that did not form carbides as a result of carburization under conditions of CO formation [66]. It has been established that FeC_x_ species are formed by the conversion of surface layers of the Fe_3_O_4_ phase [67]. In addition, in the presence of a carburizing environment (CO_2_/H_2_), Fe^0^ species could be quickly transformed into hexagonal iron carbides [68,69]. Fe_2_O_3_ as well as Fe are not active in Fischer–Tropsch synthesis and hydrocarbon formation starts only after partial carburization [66,67,70]. Thus, the increased activity of some samples at low temperatures is indirect evidence of Fe_3_O_4_, Fe, and carbide phase formation during the reaction. It is stated in the literature that oxide and carbide nanoparticles of Fe located in close proximity to each other provide active sites for RWGS and FTS [71,72,73]. At the same time, CO_2_ conversion and selectivity to hydrocarbons depend on the ratio of Fe_3_O_4_ and Fe_5_C_2_ phases [74].

**Figure 9:**
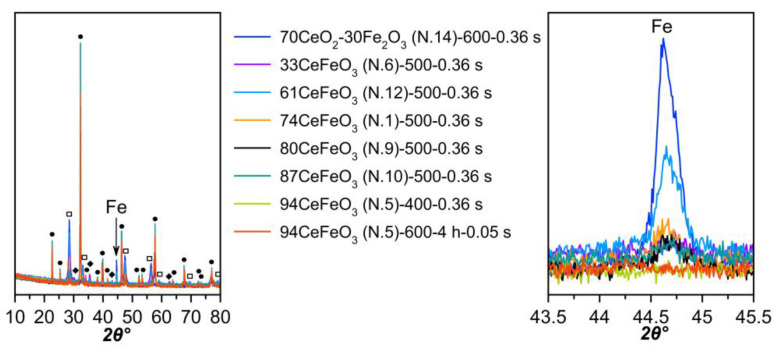
X-ray diffraction patterns of spent catalysts (the legend indicates the tested temperature, residence time, and TOS). ●–o-CeFeO_3_ (JCPDS card no. 00-022-0166); □–cubic CeO_2_ (JCPDS card no. 01-075-8371); ◆–γ-Fe_2_O_3_ (JCPDS card no. 00-039-1346).

XRD did not detect iron carbide in the spent catalysts (Figure 9), which is probably due to its small amount, in line with low selectivity to hydrocarbons. The only new phase is metallic iron, which appeared in the XRD patterns of the samples tested at 500 °C and a high contact time (0.36 s). The Ce–Fe oxide system has the highest metallic iron content after the reaction. In [75], an increase in the amount of surface Fe^0^ species in the freshly reduced catalysts led to a linear decrease in methane yield.

On the other hand, a contribution to the increase in activity is also possible due to the presence of cerium. Compared to CeO_2_ and Fe_2_O_3_, mixed samples of CeO_2_–Fe_2_O_3_ displayed increased activity, especially 20 wt% CeO_2_ [51]. Moreover, a combination of both oxides efficiently suppresses the sintering and improves stability in CO_2_ reduction to CO by chemical looping. The effect of ceria can also be associated with the formation of a Ce–Fe oxide solid solution that increases the specific surface area and, possibly, increases the electron density on Fe atoms in active carbide phases, acting as a main electronegative promoter [76]. Therefore, the catalytic performance depends both on the activity of each active phase and on their spatial distribution, and close contact between them.

The samples that are most active at low temperatures have in common the utilization of ammonium nitrate as an additive to the oxidizer. The flash pyrolysis of NH_4_NO_3_ is known to yield N_2_, H_2_O, and O_2_, excluding a possibility of NO_x_ formation [77]. An increased gas formation could push the oxide phases to the surface of ferrite and increase their reactivity.

**Figure 10:**
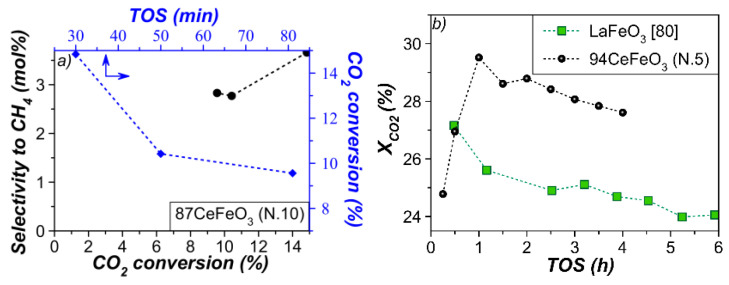
Dependence of CO_2_ conversion and selectivity to CH_4_ on time-on-stream (TOS) at (**a**) 400 °C, H_2_:CO_2_:N_2_ = 3:1:1, WHSV = 10,000 mL·g^–1^·h^–1^; and (**b**) 600 °C, H_2_:CO_2_:N_2_ = 1:1:1, WHSV = 72,000 mL·g^–1^·h^–1^.

Dependences of CO_2_ conversion on time-on-stream and CH_4_ selectivity on conversion for the material active in the FTS reaction are presented in Figure 10a. It can be seen that there is a synchronous decrease in conversion and selectivity. The main deactivation factor is usually considered to be coke deposits as a result of secondary reactions [78]. In addition, it is clearly seen from Figure 10a that non-zero selectivity to CH_4_ is anticipated at a very close to zero conversion of CO_2_, which implies that CO_2_ is directly (without intermediate CO formation) hydrogenated to CH_4_ [79].

To provide a final comparison of the samples obtained in this study with other perovskite-based catalysts previously applied for CO_2_ hydrogenation, their activity was compared under the same reaction conditions. In [80], LaFeO_3_ was tested at 600 °C, WHSV of 72,000 mL·g^–1^·h^–1^, and H_2_/CO_2_ ratio of 1. The sample containing 94 wt% CeFeO_3_ perovskite demonstrated a higher conversion with 100 mol% CO selectivity compared to LaFeO_3_ (Figure 10b). In the first hour of the reaction, an induction period of catalyst activation is observed, during which the activity increases from 24.8 to 29.5%, due to the residual reduction of iron oxides. The deactivation rate constant (*k_d_*) was the same as for LaFeO_3_ (0.02 h^–1^). In fact, in this case, deactivation is slower than in the case of a commercial catalyst (*k_d_ =* ~0.13 h^–1^, 5 h of TOS [81]).

It was suggested that the activity of LaFeO_3_ is due to the abundance of oxygen vacancies, which contribute to CO_2_ activation [80]. For the CeFeO_3_ catalyst, the synergy between the phases is explained by the creation of a large amount of oxygen vacancies in the presence of CeFeO_3_ and CeO_2_ stimulating the migration of oxygen ions, while iron species on the surface promote a pathway of the oxygen spillover from subsurface to surface and improve lattice oxygen transfer [82,83]. CO_2_ adsorption and dissociation on CeO_2_ surface defects followed by the formation of carbonates and formates are also often considered as the reaction mechanism for the RWGS reaction over CeO_2_-supported catalysts [84,85]. The presence of a large amount of Fe^0^ in the Fe_2_O_3_–CeO_2_ composite and the oxygen deficiency of CeFeO_3_ obtained using NH_4_NO_3_ can contribute to a change in the reaction mechanism. Both systems are generally characterized by the redox mechanism; however, for defective CeFeO3, the role of associative mechanisms seems to also increase at 300–400 °C [85].

CO_2_ hydrogenation for the catalyst and some materials reported in the literature are presented in Table 5. The CeFeO_3_-based catalyst exhibited a similar or even higher CO_2_ hydrogenation rate compared to some other recently reported materials, including the commercial catalyst Cu/ZnO/Al_2_O_3_.

It was also considered how much carbon dioxide emissions are generated during the synthesis of the materials compared to the catalytic process. The mass balance is presented in Table S4. For the calculations, a reaction equation was used giving the maximum formation of perovskite, that is, the one corresponding to glycine and NH_4_NO_3_ as reagents. Excluding ammonium nitrate, the fuel-to-oxidizer ratio is 1.5, meaning that because of a slight excess of fuel more gases are released.

During the synthesis of 1 g of perovskite, ca. 0.9 L of CO_2_ is released. Therefore, there is less carbon dioxide per 300 and 100 mg of the catalyst (shown in Table S5). Based on the conditions of the experiments carried out in this study, the time to reach the conversion corresponding to released amounts of CO_2_ during the synthesis of the perovskite was calculated. Such time depends on conversion and WHSV. On the other hand, the catalyst can operate for a long time both with or without regeneration. Therefore, it can be concluded that the amount of reacted carbon dioxide through its hydrogenation exceeds the amount of CO_2_ formed during the synthesis of perovskites.

## 4. Conclusions

In this study, solution combustion synthesis has been successfully applied for the preparation of CeFeO_3_ perovskite-based catalysts, which were investigated for the first time in CO_2_ hydrogenation. Hydrogenation of CO_2_ from industrial exhaust gases via the reverse water–gas shift reaction (RWGS) to CO represents one of the most promising CO_2_ utilization processes because CO can be used in the downstream Fischer–Tropsch (FTS) reaction and other applications. The driving force for the development of new systems for this process is the low thermal stability of industrial copper-based catalysts at high temperatures and, therefore, their rapid deactivation because of sintering and reoxidation. CeFeO_3_, in turn, is highly stable under reducing conditions and, due to its defective structure, has the potential to be used in CO_2_ hydrogenation.

The solution combustion synthesis method represents an alternative to the traditional techniques due to its short synthesis time, low cost, and energy savings, while the main drawback is associated with the formation of pollutant gases, NH_3_ or NO_x_. In this respect, SCS is similar to other methods when nitrate precursors are used. To achieve the highest yield of cerium orthoferrite, several fuels were used, such as glycine, urea, and urotropine, as well as such additives as glucose and ammonium nitrate. NH_4_NO_3_ prevents oxidation of Ce(III) to Ce(IV), which is usually an undesirable side reaction. In combination with glycine, NH_4_NO_3_ makes it possible to obtain the highest yield of cerium orthoferrite (94 wt%). Depending on the synthesis conditions, the combustion products had different phase compositions, crystallinity, specific surface area, and reducibility, the effect of which was evaluated in CO_2_ hydrogenation.

Various CeFeO_3_–CeO_2_–Fe_2_O_3_ systems containing from 0 to 94 wt% CeFeO_3_ have shown that RWGS reaction is dominant in the CO_2_ hydrogenation process. At the same time, it was found that samples prepared using ammonium nitrate are more active at temperatures of 300–400 °C and form hydrocarbons (CH_4_, C_2_H_4_, C_2_H_6_, and C_3_H_8_) in addition to CO. The best catalytic performance in CO_2_ hydrogenation at low temperatures and the H_2_:CO_2_ ratio of 3:1 was obtained with a sample containing 33% CeFeO_3_ prepared using glycine and ammonium nitrate.

At 600 °C and the H_2_:CO_2_ ratio of 1:1, the CeFeO_3_-based catalyst exhibits higher activity than another member of the rare-earth orthoferrite class LaFeO_3_. Activity can be enhanced by the presence of unbound cerium and iron oxides, due to the fact that Ce–Fe perovskite and CeO_2_ phases can produce more oxygen vacancies and promote migration of the oxygen ion, while surface iron particles facilitate the oxygen spillover from subsurface to surface.

## Figures and Tables

**Table 1:** Data on the phase composition and crystallinity of the synthesized materials according to the refinement by the Rietveld method.

Sample N	Fuel	n(AN)/ n(MeN)	φ	Phase Composition According Rietveld/Corrected ^1^, wt%			D (1), nm	D (2), nm	D (3), nm	R_wp_/R_e_
				CeFeO_3_	CeO_2_	Fe_2_O_3_				
1	Glycine	–	1	81/74	19/18	0/8	67.5	4.7	–	–
2	Glycine	–	1.25	78	12	10	66.0	7.5	6.7	1.14
3	Glycine	–	1.4	95/93	5/5	0/2	61.3	25.0	–	1.16
4	Glycine	–	1.5	87/82	13/12	0/6	56.8	9.0	–	–
5	Glycine	1.5	1.25	96/94	4/4	0/2	69.1	15.6	–	1.13
6	Glycine	1.5	1.75	33	47	20	32.7	14.8	14.7	1.03
7	Glycine + glucose	–	1.4	63	23	14	52.0	19.4	14.3	–
8	Urea	–	2.5	52	28	20	68.6	5.8	4.0	–
9	Urea	–	3.5	80	13	7	72.0	4.7	6.3	1.09
10	Urea	1.5	2.9	91/87	9/7	0/4	66.5	22.0	–	1.17
11	Urotropine	–	1.5	48	33	19	75.1	22.1	15.8	–
12	Urotropine	–	2	61	23	16	60.0	16.4	7.1	1.09

^1^ Taking into account amorphous iron oxide according to the equation wt%(Fe_2_O_3_) = wt%(CeO_2_)/2.156, where 2.156 is CeO_2_/Fe_2_O_3_ ratio obtained at oxidation of CeFeO_3_; AN–ammonium nitrate; MeN–cerium and iron nitrates; φ–the fuel-to-oxidizer ratio; D–the mean crystallite size; 1–CeFeO_3_; 2–CeO_2_; 3–γ-Fe_2_O_3_; R_wp_/R_e_ characterizes goodness of fit, if the squared value is equal to one or constant the refinement procedure is complete.

**Table 2:** The mean size of CeO_2_ crystallites (D) and total weight loss for Ce-Fe oxide systems obtained by the SCS method using various types of fuel and the fuel-to-oxidizer ratio.

Sample N.	Fuel	φ	D (CeO_2_), nm	Total Weight Loss According to DTA, wt%
13	Glycine + glucose	2.2	1.9	29.6
14	Urea	1	5.7	4.6
15	Urea	1.5	5.0	4.4
16	Urotropine	1	6.5	5.5
17	Urotropine	6	4.7	–

**Table 3:** Textural characteristics of the as-prepared samples produced by solution combustion synthesis using different fuels, additives, and fuel-to-oxidizer ratios (φ).

Sample N.	Fuel, Additive, φ	Mass Fraction of CeFeO_3_	S_BET_, m^2^/g	ΣVpore, cm^3^/g	Average Pore Size, nm
3	Glycine, 1.4	0.93	3	0.01	16.5
5	Glycine, NH_4_NO_3_, 1.25	0.94	2	0.01	15.0
7	Glycine, glucose, 1.4	0.63	8	0.12	60.5
9	Urea, 3.5	0.80	3	0.05	59.0
12	Urotropine, 2	0.61	4	0.05	47.5
15	Urea, 1.5	0	81	0.13	6.3

**Table 4:** H_2_-TPR results and cell parameters for studied in TPR samples.

Sample	Unit-Cell Volume (CeFeO_3_), Å^3^	Lattice Parameter (CeO_2_), Å	Theoretical H_2_ Uptake ^1^, mmol/g			H_2_ Uptake According to TPR, mmol/g			
			CeO_2_	Fe_2_O_3_	Total	Area 1 (α + ε)	Area 2 (β + δ)	Area 3 (γ + ζ)	Total
N.15	–	5.376	2.05	6.15	8.20	3.77	5.42	0.7	9.89
N.6	240.120	5.406	1.37	3.76	5.12	1.37	4.49	1.98	7.84
N.11	239.833	5.408	0.96	3.57	4.53	1.13	2.82	2.82	6.77
N.4	239.511	5.402	0.35	1.13	1.48	0.73	1.29	5.23	7.25
N.5	239.930	5.396	0.12	0.38	0.49	0.62	0.78	5.69	7.09

^1^ Assuming a complete reduction up to Ce_2_O_3_ and Fe^0^, respectively.

**Table 5:** Comparison of catalyst selectivity and CO_2_ conversion rate, P = 1 atm, T = 600 °C.

Catalyst	H_2_:CO_2_	WHSV, mL·g^–1^·h^–1^	CO Selectivity, %	Rate (mmol CO_2_/(g_cat_·s))	Ref.
94CeFeO_3_–CeO_2_–Fe_2_O_3_	1:1	72,000	100	0.064	This work
5Cu/LaFeO_3_	1:1	72,000	100	0.10	[80]
Commercial Cu/ZnO/Al_2_O_3_	2:1	300,000	100	0.076	[81]
CuAl_2_O_4_	2:1	300,000	100	0.005	[81]
Cu−Fe/SiO_2_	1:1	120,000	no data	0.037	[86]
5Fe/CeO_2_	1:1	600,000	no data	0.389	[87]

## Supplementary Materials

The following supporting information can be downloaded at: https://www.mdpi.com/article/10.3390/ma15227970/s1, Figure S1: Total weight change during heating in air from 25 to 800 °C for the as-prepared samples produced by SCS using different fuels, additives and fuel-to-oxidizer ratios; Figure S2: XRD data for Ce–Fe oxide systems obtained by the SCS method using various types of fuel and fuel-to-oxidizer ratios; Figure S3: Dependence of the rate for a heterogeneous catalytic reaction on temperature; Table S1: Assignment of major absorption IR spectra peaks for the as-prepared samples produced by solution combustion synthesis using different fuels; Table S2 Data on the phase composition, crystallinity and reproducibility of the synthesized materials according to the refinement by the Rietveld method; Table S3: Influence of additions (glucose, NH_4_NO_3_) on glycine(or urea)-nitrate solution combustion process and the obtained SCS products; Table S4: The mass balance of combustion process; Table S5: Calculation of the volume of released CO_2_ and time for its conversion.
