# Supplementary material for: CeFeO3–CeO2–Fe2O3 Systems: Synthesis by Solution Combustion Method and Catalytic Performance in CO2 Hydrogenation
Source: Materials (Basel). 2022 Nov 11;15(22):7970. doi: 10.3390/ma15227970 (PMC9696793; doi:10.3390/ma15227970)
Supplement: Supplementary file 1 [file materials-15-07970-s001.zip › materials-2014691-supplementary.pdf]

Supplementary Material

CeFeO$_3$–CeO$_2$–Fe$_2$O$_3$ systems: synthesis by solution combustion method and catalytic performance in CO$_2$ hydrogenation

Anna N. Matveyeva[a], Shamil O. Omarov[a], Marianna A. Gavrilova[a], Dmitry A. Dmitry A. Sladkovskiy[b], and Dmitry Yu. Murzin[*c]*

[a]Laboratory of Materials and Processes for Hydrogen Energy, Ioffe Institute, Politekhnicheskaya ul. 28, St. Petersburg 194021, Russia

[b]Laboratory of Catalytic Technologies, St. Petersburg State Institute of Technology (Technical University), Moskovskiy pr. 26, St. Petersburg 190013, Russia

[c]Laboratory of Industrial Chemistry and Reaction Engineering, Åbo Akademi University, Henriksgatan 2, Turku/Åbo 20500, Finland

*e-mail: dmurzin@abo.fi

Chemical equations with fuel to calculate the amount of reagents

Glycine

Total valency of glycine: $C_2H_5NO_2 = 4·2 + 1·5 – 2·2 = +9$

Total valency of oxidizers: $Ce(NO_3)_3 = 3 – 2·3·3 = –15$; $Fe(NO_3)_3 = 3 – 2·3·3 = –15$

1) *φ=1:* $3Ce(NO_3)_3(aq) + 3Fe(NO_3)_3(aq) + 10C_2H_5NO_2 \rightarrow 3CeFeO_3 + 20CO_2 + 14N_2 + (25H_2O)$

2) *φ=1.25:* $24Ce(NO_3)_3(aq) + 24Fe(NO_3)_3(aq) + 100C_2H_5NO_2 + 45O_2 \rightarrow 24CeFeO_3 + 200CO_2 + 122N_2 + (250H_2O)$

3) *φ=1.4:* $3Ce(NO_3)_3(aq) + 3Fe(NO_3)_3(aq) + 14C_2H_5NO_2 + 9O_2 \rightarrow 3CeFeO_3 + 28CO_2 + 16N_2 + (35H_2O)$

4) *φ=1.5:* $4Ce(NO_3)_3(aq) + 4Fe(NO_3)_3(aq) + 20C_2H_5NO_2 + 15O_2 \rightarrow 4CeFeO_3 + 40CO_2 + 22N_2 + (50H_2O)$

Urea

Total valency of urea: $(NH_2)_2CO = 1·4 + 4 – 2 = +6$

5) *φ=1:* $Ce(NO_3)_3(aq) + Fe(NO_3)_3(aq) + 5(NH_2)_2CO \rightarrow CeFeO_3 + 5CO_2 + 8N_2 + (10H_2O)$

6) *φ=1.5:* $4Ce(NO_3)_3(aq) + 4Fe(NO_3)_3(aq) + 30(NH_2)_2CO + 15O_2 \rightarrow 4CeFeO_3 + 30CO_2 + 42N_2 + (60H_2O)$

7) *φ=2.5:* $4Ce(NO_3)_3(aq) + 4Fe(NO_3)_3(aq) + 50(NH_2)_2CO + 25O_2 \rightarrow 4CeFeO_3 + 30CO_2 + 62N_2 + (100H_2O)$

8) *φ=3.5:* $4Ce(NO_3)_3(aq) + 4Fe(NO_3)_3(aq) + 70(NH_2)_2CO + 75O_2 \rightarrow 4CeFeO_3 + 70CO_2 + 82N_2 + (140H_2O)$

Urotropine

Total valency of urotropine: $C_6H_{12}N_4 = 4\cdot6 + 1\cdot12 = +36$

9) *φ=1:* $6Ce(NO_3)_3(aq) + 6Fe(NO_3)_3(aq) + 5C_6H_{12}N_4 \rightarrow 6CeFeO_3 + 30CO_2 + 28N_2 + (30H_2O)$

10) *φ=1.5:* $4Ce(NO_3)_3(aq) + 4Fe(NO_3)_3(aq) + 5C_6H_{12}N_4 + 15O_2 \rightarrow 4CeFeO_3 + 30CO_2 + 22N_2 + (30H_2O)$

11) *φ=2:* $3Ce(NO_3)_3(aq) + 3Fe(NO_3)_3(aq) + 5C_6H_{12}N_4 + 24O_2 \rightarrow 4CeFeO_3 + 30CO_2 + 19N_2 + (30H_2O)$

12) *φ=6:* $2Ce(NO_3)_3(aq) + 2Fe(NO_3)_3(aq) + 10C_6H_{12}N_4 + 75O_2 = 2CeFeO_3 + 60CO_2 + 26N_2 + (60H_2O)$

Glycine and NH_4NO_3

Total valency of oxidizers: $NH_4NO_3 = 1\cdot4 – 2\cdot3 = –2$

13) *φ=1.25:* $4Ce(NO_3)_3(aq) + 4Fe(NO_3)_3(aq) + 20C_2H_5NO_2 + 12NH_4NO_3 + 9O_2 \rightarrow 4CeFeO_3 + 40CO_2 + 34N_2 + (74H_2O)$

14) *φ=1.75:* $4Ce(NO_3)_3(aq) + 4Fe(NO_3)_3(aq) + 28C_2H_5NO_2 + 12NH_4NO_3 + 27O_2 \rightarrow 4CeFeO_3 + 56CO_2 + 38N_2 + (94H_2O)$

Glycine and glucose

Total valency of glucose: $C_6H_{12}O_6 = 4\cdot6 + 1\cdot12 – 2\cdot6 = +24$

15) *φ=1.4:* $9Ce(NO_3)_3(aq) + 9Fe(NO_3)_3(aq) + 30C_2H_5NO_2 + 5C_6H_{12}O_6 + 30O_2 \rightarrow 9CeFeO_3 + 90CO_2 + 42N_2 + (105H_2O)$

16) *φ=2.2:* $12Ce(NO_3)_3(aq) + 12Fe(NO_3)_3(aq) + 60C_2H_5NO_2 + 10C_6H_{12}O_6 + 105O_2 \rightarrow 12CeFeO_3 + 180CO_2 + 66N_2 + (210H_2O)$

Urea and NH_4NO_3

17) *φ=2.9:* $4Ce(NO_3)_3(aq) + 4Fe(NO_3)_3(aq) + 70(NH_2)_2CO + 12NH_4NO_3 + 69O_2 \rightarrow 4CeFeO_3 + 70CO_2 + 94N_2 + (164H_2O)$

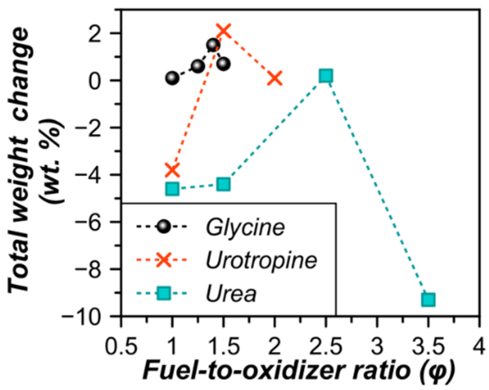

Figure S1. Total weight change during heating in air from 25 to 800 °C for the as-prepared samples produced by SCS using different fuels, additives and fuel-to-oxidizer ratios.

Table S1. Assignment of major absorption IR spectra peaks for the as-prepared samples produced by solution combustion synthesis using different fuels.

Wavenumber, cm^{-1}	Assignment	Substance
3727	ν(OH)	adsorbed H_2O
3343–3453	ν_s, ν_{as} (H_2O)	
3212–3430	ν_s, ν_{as} (NH_3)	unreacted fuel
2959	ν(CH)	
2920–2924	ν_{as}(CH_2)	
2852–2854	ν_s(CH_2)	
2100–2105	ν(-C≡C-)	
1709	ν(C=O)	
1664–1667	ν_{as}(COO), ν(C=O)	
1619–1621	β_{as}(NH_3)	
1446	δ(CH_2)	
1383–1385	ν_s(NO_3^-)	unreacted nitrate groups
1115–1156	ρ(NH_3)	unreacted fuel
548–552, 404–406	ν(FeO)	$CeFeO_3$
Symbols for vibrations: ν, stretching; δ, deformation; β, bending; ρ, rocking; as, antisymmetric; s, symmetric.		

Table S2. Data on the phase composition, crystallinity and reproducibility of the synthesized materials according to the refinement by the Rietveld method.

Sample N	Fuel	n(AN)/ n(MeN)	φ	Phase composition (Rietveld refinement), wt%			D (1), nm	D (2), nm	D (3), nm	R_{wp}/ R_e
				$CeFeO_3$	CeO_2	γ-Fe_2O_3				
1	Glycine	–	1	81	19	0	67.5	4.7	–	–
2		–	1.25	78	12	10	66.0	7.5	6.7	1.14
3-1		–	1.4	95	5	0	61.3	25.0	–	1.16
3-2				93	7	0	64.0	14.5	–	1.11
4		–	1.5	87	13	0	56.8	9.0	–	–
5-1		1.5	1.25	96	4	0	69.1	15.6	–	1.13
5-2				94	6	0	72.0	18.6	–	1.13
6			1.75	33	47	20	32.7	14.8	14.7	1.03
7	Glycine+ glucose	–	1.4	63	23	14	52.0	19.4	14.3	–
8	Urea	–	2.5	52	28	20	68.6	5.8	4.0	–
9-1		–	3.5	80	13	7	72.0	4.7	6.3	1.09
9-2				84	16	0	60.4	4.2	–	1.10
10-1		1.5	2.9	91	9	0	66.5	22.0	–	1.17
10-2				92	8	0	72.0	13.1	–	1.11
11-1	Urotropine	–	1.5	48	33	19	75.1	22.1	15.8	–
11-2				42	42	16	74.5	12.5	25.3	1.05
12		–	2	61	23	16	60.0	16.4	7.1	1.09

Note: AN – ammonium nitrate; MeN – cerium and iron nitrates; φ – fuel-to-oxidizer ratio; 1 – $CeFeO_3$; 2 – CeO_2; 3 – γ-Fe_2O_3; the ratio of the weighted (R_{wp}) and expected (R_e) R-factors characterizes goodness of fit, if the squared value is equal to one or constant the refinement procedure is complete.

Table S3. Influence of additions (glucose, NH_4NO_3) on glycine(or urea)-nitrate solution combustion process and the obtained SCS products.

Fuel, addition	Glycine, glucose	Glycine, NH_4NO_3	Glycine, NH_4NO_3	Urea, NH_4NO
φ	1.4	1.25	1.75	2.9
Course of the reaction	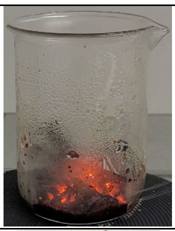	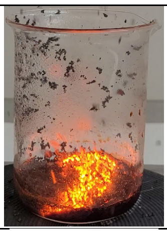	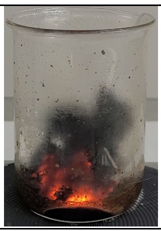	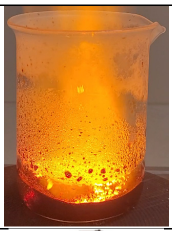
Macromorphology of the obtained materials	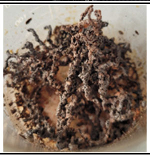	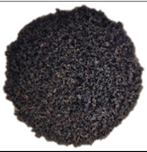	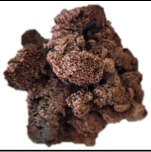	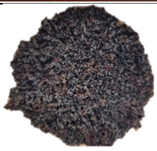
Total Δm during heating up to 800 ºC in air, wt%	+0.9	+1.2	–1.2	–1.0
Weight gain, wt%	1.6	2.5	0.5	2.5

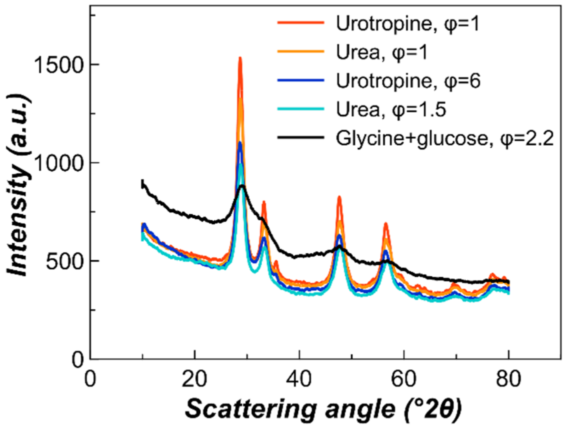

Figure S2. XRD data for Ce-Fe oxide systems obtained by the SCS method using various types of fuel and fuel-to-oxidizer ratios.

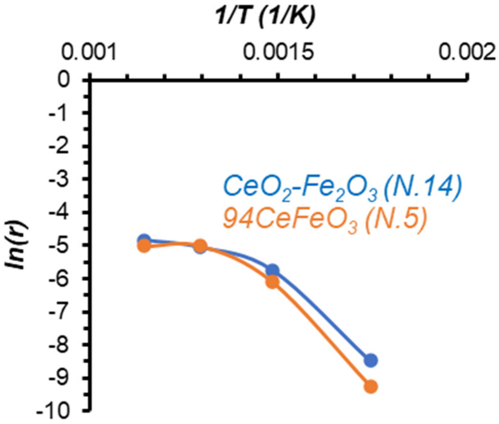

Figure S3. Dependence of the rate for a heterogeneous catalytic reaction on temperature.

Mass balance

<u>*Given:*</u>

φ=1.25: $4Ce(NO_3)_3 \cdot 6H_2O + 4Fe(NO_3)_3 \cdot 9H_2O + 20C_2H_5NO_2 + 12NH_4NO_3 + 9O_2 \rightarrow 4CeFeO_3 + 40CO_2 + 34N_2 + (134H_2O)$

$m(CeFeO_3) = 1$ g

$V(CO_2)$ – ?

<u>*Solution:*</u>

Table S4. The mass balance of combustion process

Compound	M, g/mol	Input		Output	
		m_1, g	N_1, mol	m_2, g	N_2, mol
$Ce(NO_3)_3 \cdot 6H_2O$	434.2	1.780	0.004	0	0

$Fe(NO_3)_3·9H_2O$	404.0	1.656	0.004	0	0
$C_2H_5NO_2$	75.1	1.539	0.020	0	0
NH_4NO_3	80.0	0.984	0.012	0	0
O_2	32.0	0.295	0.009	0	0
$CeFeO_3$	244.0	0	0	1.000	0.004
CO_2	44.0	0	0	1.804	0.041
N_2	28.0	0	0	0.976	0.035
H_2O	18.0	0	0	2.474	0.137
**Sum**	**-**	**6.254**	**0.050**	**6.254**	**0.217**

Table S5. Calculation of the volume of released CO_2 and time for its conversion

**m($CeFeO_3$), g**	**V(CO_2), L**	**H_2:CO_2 ratio**	**WHSV, mL· $g^{-1}·h^{-1}$**	**Volumetric flow rate of CO_2 for hydrogenation, L/min**	**Conversion of CO_2 at 600 °C**	**Conversion time, min**
1	0.92	-		-	-	-
0.3	0.28	3:1	10,000	0.01	0.41	11.5
0.1	0.09	1:1	72,000	0.04	0.28*	0.63

Note: *the mean value
